# Photonic effects in natural nanostructures on *Morpho cypris* and *Greta oto* butterfly wings

**DOI:** 10.1038/s41598-020-62770-w

**Published:** 2020-04-01

**Authors:** C. P. Barrera-Patiño, J. D. Vollet-Filho, R. G. Teixeira-Rosa, H. P. Quiroz, A. Dussan, N. M. Inada, V. S. Bagnato, R. R. Rey-González

**Affiliations:** 1 Universidad Nacional de Colombia Sede Bogotá, Departamento de Física, Grupo de Óptica e Información Cuántica, Ciudad Universitaria, Ed. 405 Of. 207, Bogotá, D.C. C.P. 111321 Colombia; 2 Sao Carlos Institute of Physics - University of Sao Paulo, Avenida Trabalhador São-carlense, nº 400, Parque Arnold Schimidt - CEP 13566-590, São Carlos - São Paulo – Brazil., São Carlos, SP Brazil; 3 Universidad Nacional de Colombia, Sede Bogotá, Departamento de Física, Grupo de Materiales Nanoestructurados y sus Aplicaciones, Ciudad Universitaria Ed. 404 Lab. 121C, Bogotá, D.C. C.P. 111321 Colombia; 4 Hagler Institute for Advanced Study, Texas A&M University, 400 Bizzell St, College Station, TX 77843 United States of America

**Keywords:** Nanophotonics and plasmonics, Bioinspired materials, Biophotonics

## Abstract

Photonic crystals are some of the more spectacular realizations that periodic arrays can change the behavior of electromagnetic waves. In nature, so-called structural colors appear in insects and even plants. Some species create beautiful color patterns as part of biological behavior such as reproduction or defense mechanisms as a form of biomimetics. The interaction between light and matter occurs at the surface, producing diffraction, interference and reflectance, and light transmission is possible under suitable conditions. In particular, there are two Colombian butterflies, *Morpho cypris* and *Greta oto*, that exhibit iridescence phenomena on their wings, and in this work, we relate these phenomena to the photonic effect. The experimental and theoretical approaches of the optical response visible region were studied to understand the underlying mechanism behind the light–matter interaction on the wings of these Colombian butterflies. Our results can guide the design of novel devices that use iridescence as angular filters or even for cosmetic purposes.

## Introduction

Nature is colored and attracts our attention due to its beauty and complex colors. Colors can be produced by pigmentation or by the arrangement of nanostructures. Recent studies showed that some species use this latter option to create beautiful color patterns as part of biological behavior such as reproduction or defense mechanisms as a form of mimicry^1–8^, and these tools have been perfected over millions of years in nature. In all cases, the interaction between light and matter occurs at the surface, producing diffraction, interference and reflectance phenomena. Additionally, light transmission is possible under suitable conditions. In the last decade, another optical phenomenon that has generated great interest among researchers is iridescence, which is related to the angular dependence of the observed color; there are many biological species and minerals that have this feature^9–11^. In particular, there are two Colombian butterflies, *Morpho cypris* and *Greta oto*, that exhibit an iridescent effect on their wings.

Iridescence has many feasible applications^12^, and a key advantage of structural colors over pigmented colors is the more intense coloration, especially under high light conditions. However, the physical interpretation of this phenomenon remains unclear and deserves more study. Iridescence is an effect that takes place in the visible spectrum^13,14^; thus, the interaction of light with the surfaces of these systems implies the existence of nanostructures with sizes comparable to the wavelength in the visible region. On the other hand, there are man-made structures that exhibit iridescent effects, such as TiO_2_ traces^15–17^, but total control over this property in man-made systems is not yet available. In this way, comprehension of the proper working conditions when light interacts with a butterfly’s wings is a very important step toward man-made systems that will use this property.

The *Morpho cypris* and *Greta oto* butterfly samples were provided, with authorization for their manipulation and study, by the Institute of Natural Sciences of the National University of Colombia, Bogotá campus. They belong to the teaching collection.

**Figure 1:**
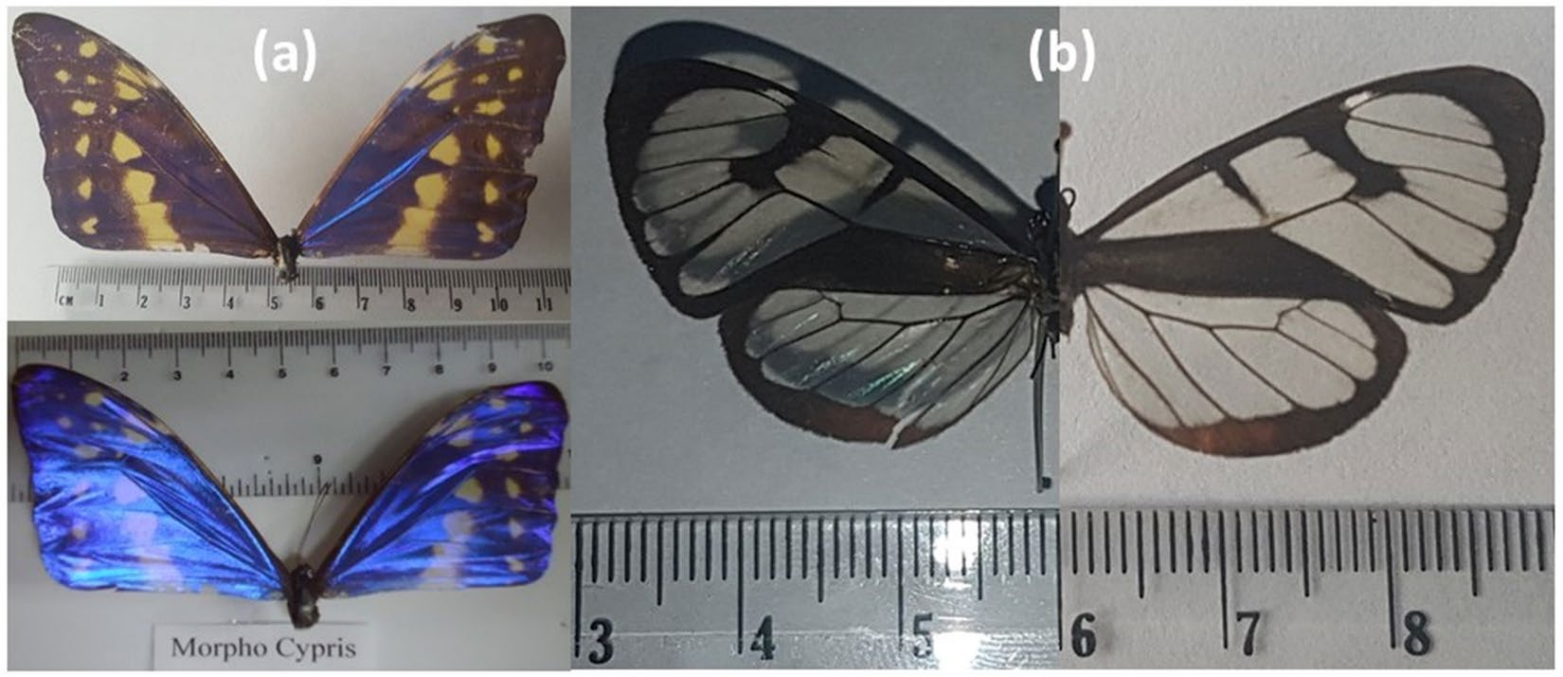
*Morpho cypris* and *Greta oto* wings with the iridescent effect. (**a**) Photographs of half of the body of *M. cypris* Colombian butterflies that show the color change. (**b**) Complete photograph of the *G. oto* Colombian butterfly. The photographs were obtained by changing the vision angle.

*M. cypris* and *G. oto* are two species of Colombian native butterflies and present colors in their wings that can be related to iridescence because these colors change with the angle of vision when the wings are observed directly (Fig. 1). These photographs were taken with a commercial cyber-shot SONY camera of 14.1 megapixels. Wing samples of *M. cypris* butterflies exhibit an intense blue color at certain viewing angles, and in others, they assume an opaque color (Fig. 1(a)). In contrast, *G. oto* butterfly wings have transparent regions relative to visible light, as shown in the left panel of Fig. 1(b), where shadows of the nerves and other structures on the wings are observable. Additionally, it is possible to observe a variety of tonalities that vary according to the angle of observation, and there is not a preferred color as in the *M. cypris* butterfly.

In this work, an experimental and theoretical approach toward the optical response in the visible region was performed to contribute to our understanding of the underlying mechanism in the light–matter interaction using wing samples from *G. oto* and *M. cypris* Colombian butterflies. For these samples, measurements were performed using scanning electron microscopy (SEM), energy-dispersive X-ray spectroscopy (EDXS), and fluorescence, reflectance and transmittance measurements for the experimental approach, while for the theoretical analysis, we modeled the arrangement of nanostructures found and determined the photonic properties of these systems.

## Results and discussion

**Figure 2:**
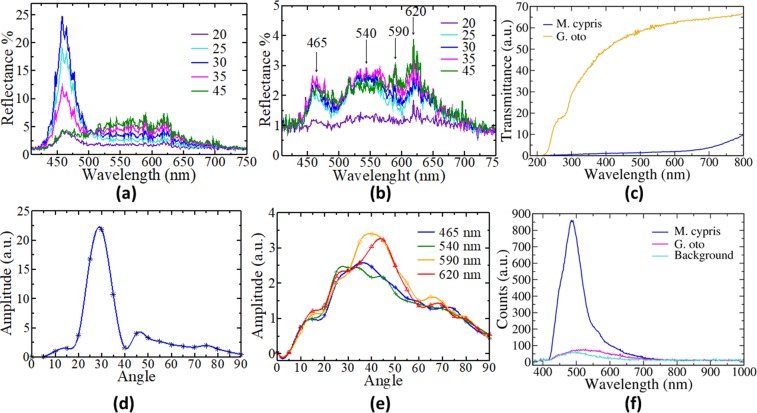
Optical measurement obtained from two species of Colombian butterflies. (**a**) Reflectance measurement as a function of the wavelength of the *M. cypris* butterfly wing sample for incidence angles between 20 and 45 grades. (**b**) The same as (**a**) for the *G. oto* butterfly wing sample. (**c**) Transmittance measurement for both samples. (**d**) Reflectance intensity as a function of the angle of incidence for a 460 nm wavelength *M*. *cypris* butterfly. (**e**) Reflectance intensity as a function of the angle of incidence for several wavelengths of the *G. oto* butterfly. (**f**) Fluorescence measurement as a function of the wavelength from every sample using a violet laser.

The optical measurements were conducted using small pieces from butterfly wings. The first step was to determine the angular dependence of reflected light on the basis of the samples. Figure 2(a,b) show the reflectance spectra as a function of the incident light wavelength for five angles of incidence for the *M. Cyprus* and *G. oto* samples, respectively. The reflection spectra, Fig. 2(a,b), were made using white light, i.e., a light source with a wide spectrum, by using a white LED that emits light over a wide range of the visible region. For the *M. cypris* butterfly wing sample, reflectance spectra were obtained for 20°, 25°, 30°, 35° and 45° incident angles, presenting a relevant peak around λ_Mc1_ = 460 *nm* (Fig. 2(a)). This peak is associated with the color blue, which is the predominant color when the wings are observed directly; see Fig. 1(a). However, for measurements at 20° and 45°, the intensity of the reflectance is very low at this point (Fig. 2(a), green and purple lines). In fact, for these angles, the intensity of reflectance is almost similar for all wavelengths. Previous works have related iridescence with periodic arrays present on the surface of wings, leaves and other biological materials, as pointed out by Melissa G Meadows, *et al*.^13^ and Paul V Braun^18^. These empirical relations have been obtained under qualitative analysis.

On the other hand, the reflectance from *G. oto* butterfly wing samples shows at least three principal peaks at approximately 465 *nm*, 590 *nm*, and 620 *nm* (Fig. 2(b)) when the spectra were obtained for similar incident angles. At 20°, the low intensity overshadows the effect of the color, which is more evident at higher angles. Similar to previous results, we observe low intensities for low angles, agreeing with the iridescent effect. An important difference is that these spectra do not exhibit a unique relevant peak, and there are some preferential colors that are reflected, even with the same intensity (see Fig. 2(b)).

Differences in the intensities of the reflection spectra can be related to the transparency, particularly the higher transparency and lower reflection intensity, as can be observed in Fig. 2(c). The *M. cypris* butterfly wings present a unique strong peak around the color blue; see Fig. 2(a). The variation in the intensity of the color blue, λ_Mc1_ = 460 *nm*, as a function of the vision angle is shown in Fig. 2(d) at the 30° angle, where the iridescence shows a greater intensity, as previously mentioned. This variation in the hue of the color blue given by the structural color is an experimental verification of what is observed with the naked eye in the wings: an iridescent phenomenon understood as the color variation with the change in the vision angle. Similarly, Fig. 2(e) shows the reflection intensity as a function of the vision angle for the wings of *G. oto* at 465 nm (blue line), 540 nm (green line), 590 nm (orange line) and 620 nm (red line).

While these results strongly reveal that the wings of *M. cypris* and *G. oto* can exhibit iridescence, it is necessary to explore whether the optical response of the wings could originate from the pigments (pigment-based color) or nanostructures (structural color)^19^. Fluorescence measurements can contribute to our understanding of this aspect^20^. Figure 2(f) shows the fluorescence results obtained using a violet laser (λ = 408 nm). The dark blue line is for the *M. cypris* sample, and the fuchsia line is for the *G. oto* sample response. The cyan line corresponds to the background (sample holder). Fluorescence measurements aim to verify that the butterfly wings of *Greta oto* behave as a transparent medium, and therefore, there is little variation with respect to the background line. These results indicate that *G. oto* butterfly wings do not have pigment-based colors, in contrast to *M. cypris* butterfly wings, which can have pigment-based colors of approximately λ_Mc3_ = 488 nm. This wavelength is different from the observed wavelength in the specular reflectance measurements, i.e., λ_Mc1_ = 460 nm. This difference occurs due to the biochemical composition not being related to the visible observations for the iridescent phenomenon. Therefore, *M. cypris* butterfly wings can exhibit both pigment-based and structural colors, while *G. oto* butterfly wings present only structural colors.

**Figure 3:**
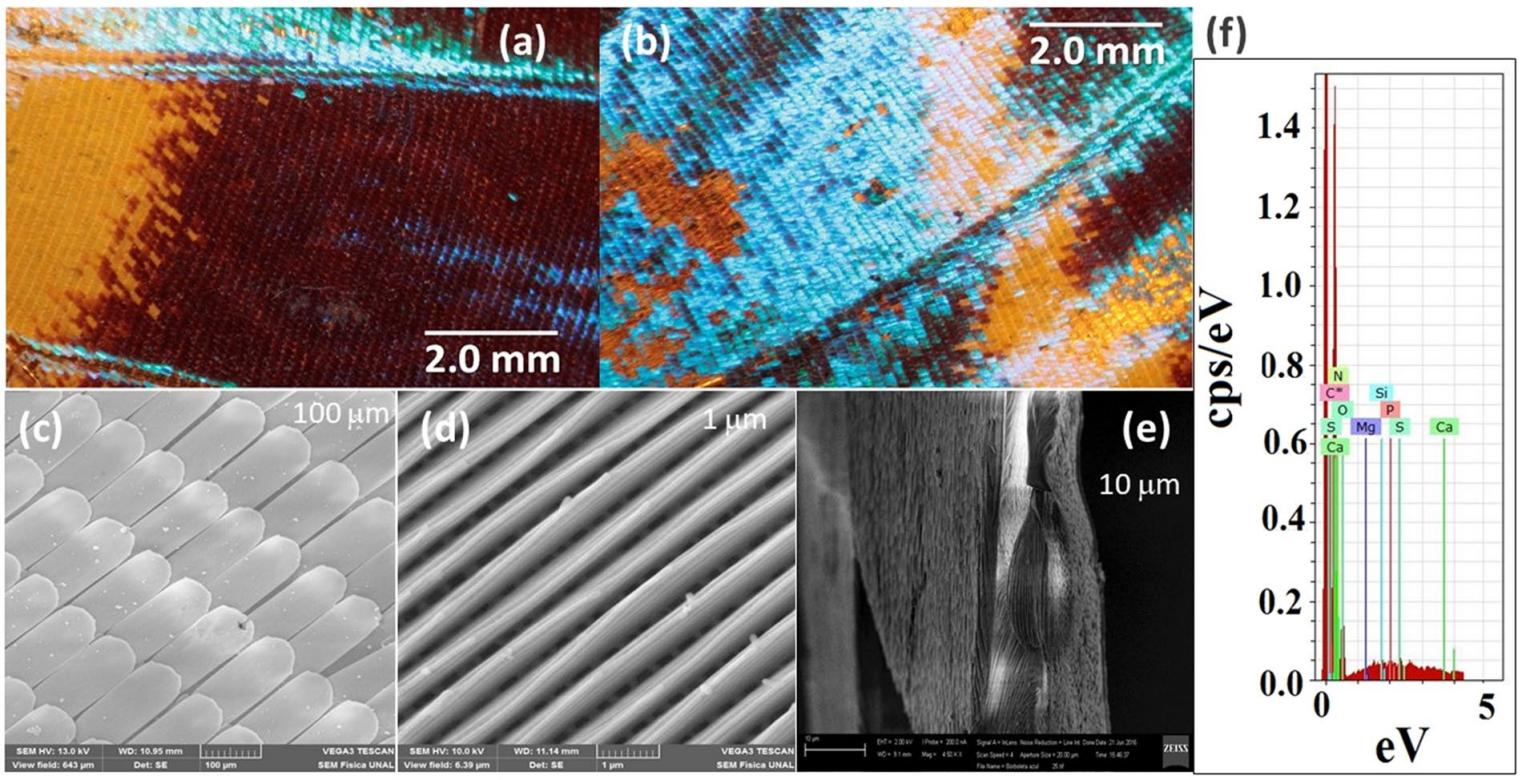
*M. cypris* butterfly surface. Photographs of the wing of the *M. cypris* Colombian butterfly taken using an optical microscope with 1x magnification at (**a**) 0° and (**b**) 60° about the normal axis. (**c**,**d**) SEM images at scales of 100 μm and 1 μm, respectively. (**e**) SEM image of the lateral disposition at 10 μm. (**f**) EDXS measurement of the wing sample.

**Figure 4:**
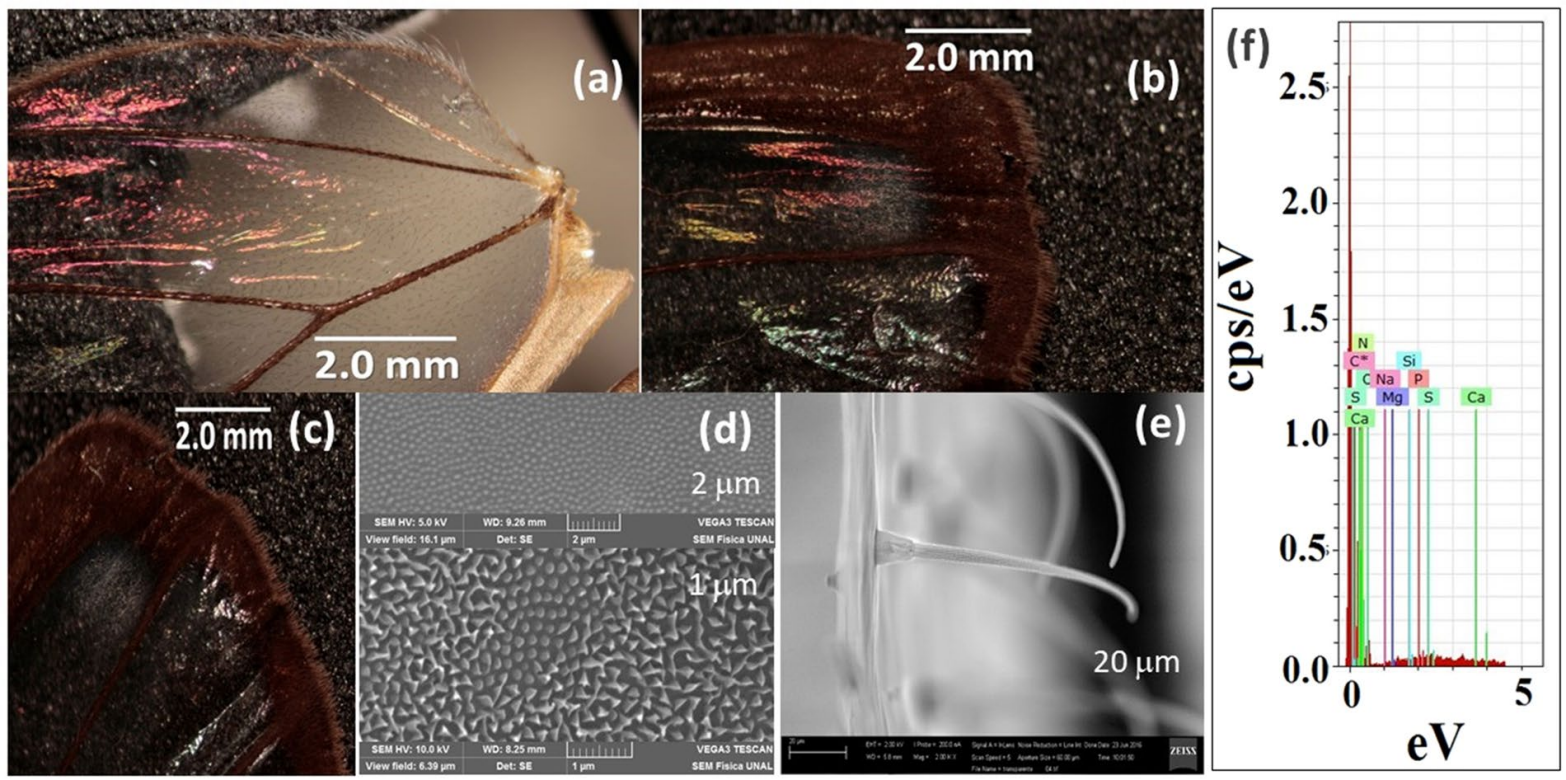
*G. oto* butterfly surface. Photograph of the wings of the Colombian butterfly *G. oto* taken using an optical microscope with 1x magnification at (**a**) 30° and (**b**) 80° about the normal axis and 15° (**c**). (**d**) Electron microscopic image at scales of 2 μm (upper panel) and 1 μm (bottom panel). (**e**) Electron microscopic image in lateral disposition at 20 μm. (**f**) EDXS measurement of the wing sample.

**Figure 5:**
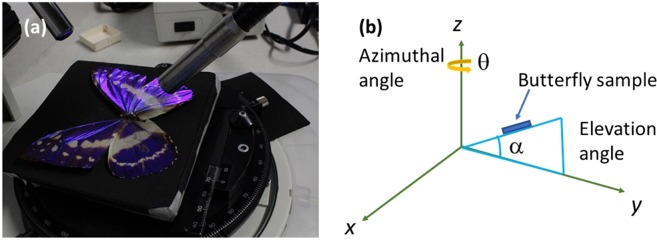
Experimental setup with an optical microscope. (**a**) Illustration of the experimental assembly of the wings in the optical microscope. (**b**) Azimuthal angle and elevation angle.

**Figure 6:**
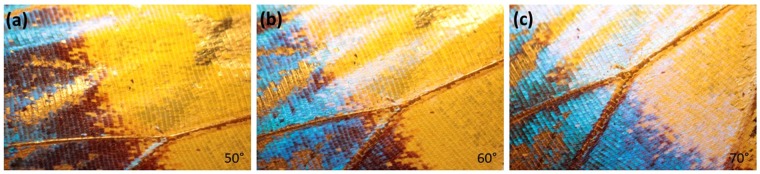
Optical microscopic images of *M. cypris* butterfly wings. Photographs of samples with azimuthal angles of 50°, 60° and 70°, 15 grades of elevation and 1x magnification.

To explore the optical phenomenon on butterfly wings, photographs were taken at different axial angles using an optical microscope with a 1x magnification without a filter or another kind of optical dispositive. The samples could rotate in their self-plane around a normal axis (axial angle) while the light source was kept fixed. Figures 3(a,b) and 4(a–c) show photographs of *M. cypris* and *G. oto* butterfly wings, where changes in the color and intensity of light as a function of the vision angle can be observed directly. The *M. cypris* sample presents a brilliant blue color at 0° and an opaque color at 60° about the normal axis; see Fig. 3(a,b). There is a change in the blue tonality in accordance with the reflectance measurements. In contrast, the *G. oto* butterfly wing has a special transparent or translucent characteristic. Photographs at 30°, 80° and 15° about the normal axis are presented in Fig. 4(a–c), respectively. Again, the change in color can be observed according to the iridescent effect that we found, and no preferable colors are observed; therefore, no preferable peak is observed. Nevertheless, the reflectance spectrum shows some reddish, blue, green or yellow tints. For these measurements, the sample is the entire wing; thus, the variation in color corresponds to the total sample, not only one region. To clarify this aspect, we show the experimental setup and the respective photographs of the *M. cypris* butterfly wings for consecutive azimuth angles of 50, 60 and 70 at 15 grades of elevation in Figs. 5 and 6 in the Experimental Methods section.

On the other hand, the wing upper surface was explored using a scanning electron microscope. From the SEM images, it was possible to observe different shapes and arrays for each of the wing samples, as shown in Figs. 3(c–e) and 4(d,e). The wing surfaces of *M. cypris* exhibit two different periodic structures: the first is a scale-like structure, and the second is a multilayer system. The scale-like structure covers all the upper surface of the wings, and each is assembled by longitudinal cuticles as a multilayer system. It was observed that the scales have a quasi-rectangular shape, (80 ± 5) μm × (140 ± 5) nm, in a 2D array (Fig. 3(c)), whereas longitudinal cuticles form a 1D pattern with a lattice parameter of (630 ± 4) nm. The last one can be understood as a multilayer system combining alternate layers of (510 ± 1) nm chitin and (120 ± 3) nm air to form a one-dimensional periodic structure. The scale-like structures have a large size in comparison with the visible wavelength; therefore, they cannot be associated directly with the iridescent effect. This effect must be related to the longitudinal cuticles and cavities (pores or holes) present on the wing surface due to the lattice parameters, which are comparable with the visible wavelengths.

In SEM micrographs of the upper surface of the wings of *G. oto*, it is possible to identify two types of structures; see Fig. 4(d,e). The larger ones are hair-like structures without a periodic distribution, whereas the others are granular spots in an apparently periodic array. Finally, it is possible to identify a hexagonal distribution at some regions of the wings. Considering the granular spots as circles, the average diameter of these nanostructures is approximately 240 ± 3 nm with a lattice parameter of 430 ± 3 nm for the 2D pattern; see Fig. 4(d). These zones are the places where the iridescent effect has been observed; see Fig. 4(a–c). Again, the size of these structures is compatible with the visible light wavelength, i.e., these structures can be responsible for the iridescence phenomenon.

Additionally, SEM images of the wing cross section were taken to try to identify other periodic arrays that can be responsible for iridescence (Figs. 3(e), 4(e)). For the cross section of *M. cypris* wings, periodic structures were not observed. In the *G. oto* wing, hair-like or capillary structures were observed. These structures have a diameter of approximately 3 μm, which is larger than the wavelength of visible light. Likewise, in Fig. 4(e), a weak second structure on the underlying surface can be observed. However, periodic arrays cannot be resolved, at least at the present magnification.

In addition, Figs. 3(f) and 4(f) show the EDXS measurements of the wing samples, which are taken to determine the chemical composition. The presence of elements such as S, C, N, O, and Si, among others, confirms chitin as the principal component. This component is characteristic of the exoskeletons of arthropods such as crabs, lobsters and insects, particularly butterflies^12^. Chitin is strong, flexible, and sometimes translucent. In the supplementary information is provided a video of iridescent effect recorded on the wings of the butterflies studied, also the data and images of measurement did from electronic microscopic image of the wings of G. oto butterfly, and a copy of the letter supplied by the Instituto de Ciencias Naturales of the Universidad Nacional de Colombia, in order to provide information on where and how we obtain the butterflies.

Recent studies relate iridescence to a photonic effect^6–9^. Photonic crystals have been extensively studied over the last decade^21–23^, and they are characterized by ordered arrays of structures with different dielectric functions^24–26^. The periodicity permits control of the light in a form similar to that of atomic potentials with electrons in insulators, semiconductors and metals. Therefore, to evaluate the periodicity in the arrays of structures found in the samples of the butterfly wings, we calculated the Fourier transform (FT) of the electron microscopic image to search for the frequency map of the images. The Fourier space map of highly ordered structures shows discrete peaks located at frequencies that can be directly related to the periodicity distance between structures. However, when images present a short-reach periodicity or when multiple periodic domains are present in the same image, the spectral density map can display less easily identifiable structures.

Another important factor is the periodicity reach, which can be evaluated by the autocorrelation length of the image. The autocorrelation function (ACF) of an image composed of identical structures will occasionally also be periodic, showing peaks in the positions related to the displacement that produce perfect overlap between different structures. When an image is not perfectly periodic, some structures can be identified in the ACF within the image autocorrelation length. Therefore, the maximum distance in which well-defined structures can be identified in the ACF can be related to the image autocorrelation length or the periodicity range of the structures.

**Figure 7:**
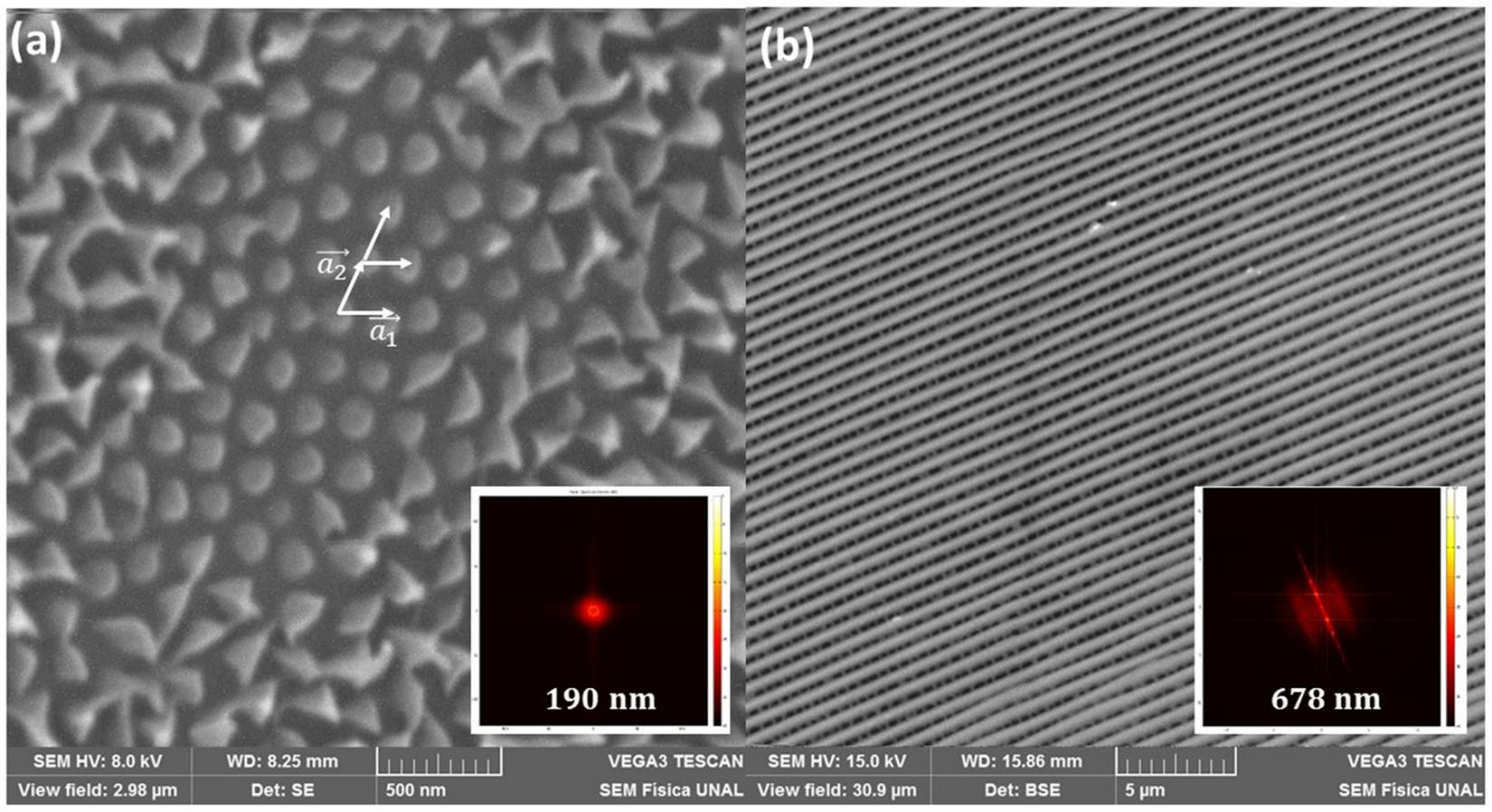
The Fourier transforms. (**a**) Electron microscopic image of wings of *G. oto* and (**b**) *M. cypris* Colombian butterflies at 0.5 and 1 μm. The inset shows the power spectrum density obtained using the FT.

The Fourier transform in the inset of Fig. 7(a) displays a ring close to the center of the power spectrum. The high-power density ring around the center indicates a predominance of spatial frequencies of approximately 5.2 μm^−1^ (period of ~190 nm), which we related to the distance between structures directly measured from the image. The Fourier map shows a homogeneous angular distribution, which means that the structures observed in the electron microscopic image present a uniform angular distribution (i.e., in a circle-like pattern) and are uniformly spaced in repeating clusters.

The autocorrelation function of Fig. 7(a) agrees with the results obtained by the Fourier transform. The central spot (no displacement) size is related to the mean size of the wing structures. The radius of the first ring surrounding the central peak is approximately 200 μm, which relates to the displacement required to make the structures overlap with their first neighbors. The existence of a ripple with approximately the same size and periodicity in the whole image is indicative that there is a long autocorrelation length on those structures.

The power spectrum density of the SEM images in the inset of Fig. 7(b) shows very narrow and well-defined peaks located along the orthogonal axis relative to the fiber-like structures. The first-order frequency relates to a spatial periodicity of 678 nm, which confirms what can be directly measured on the image. The Fourier transform also proves that this sample is indeed periodic with a very well-defined direction.

**Figure 8:**
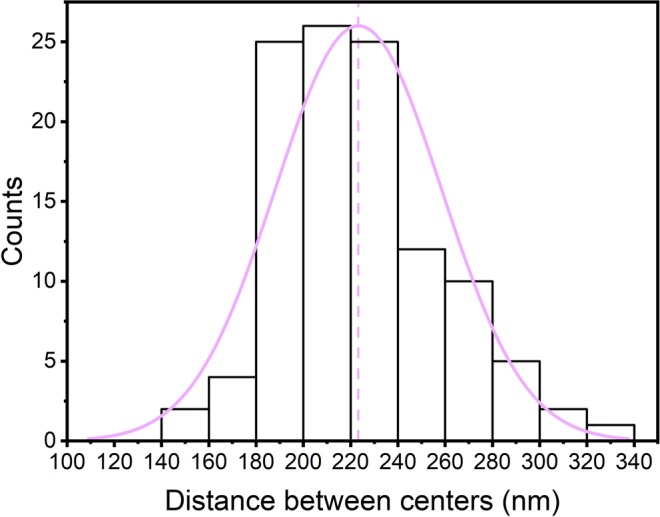
Distance between centers of structural elements from electron microscopic image of the wings of the *G. oto* butterfly. Distribution of the measurements of the distance between the centers of the structural elements inside the sample wings ((223 ± 35) nm); the pink line shows the median value of the distance between the centers.

**Figure 9:**
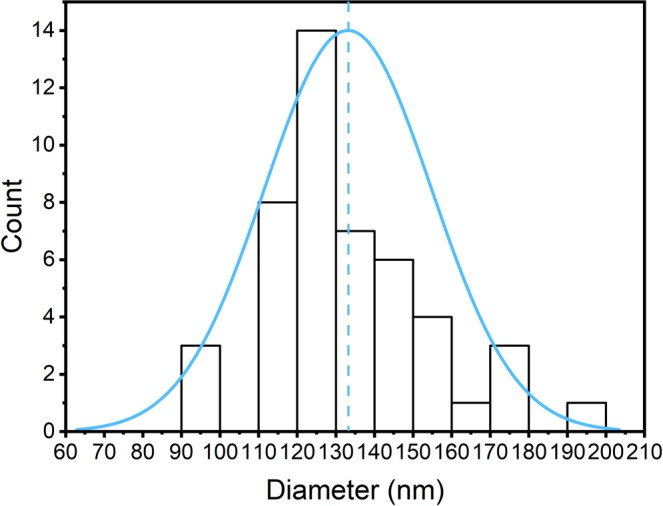
Measurements of the diameter of structural elements from electron microscopic images of the wings of the *G. oto* butterfly. Distribution of the measurements of the diameters of the structural elements inside the sample wings ((133 ± 22) nm); the blue line shows the median value of the diameter.

To analyze the degree of periodicity involved in the structure, we carry out a measurement that relates the average distance between the structural elements, as well as their average size, along the sample. The distribution of these grains reveals the degree of periodicity or structural randomness (Figs. 8, 9).

**Figure 10:**
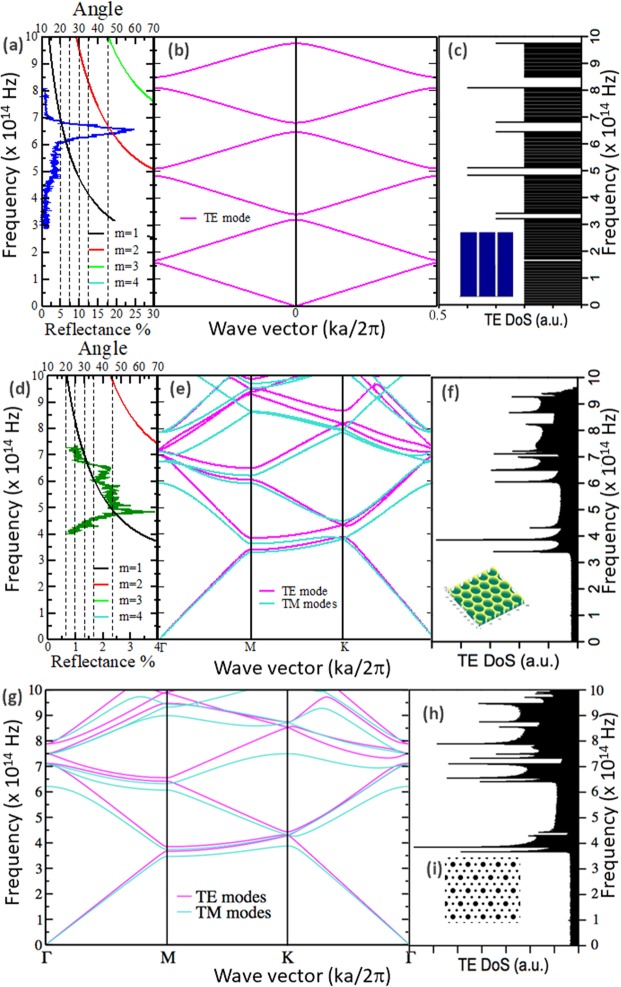
Bragg curves, reflectance, PBG and PDoS of the butterfly wings. (**a**) Reflectance spectrum of the *M. cypris* butterfly wing sample at 30° and Bragg curves for m = 1 (black line), m = 2 (red line) and m = 3 (green line). (**b**) Photonic dispersion relation for the 1D model. (**c**) Photonic density of states (PDoS) for the TE mode for the PBG shown in (**b**). (**d**) Reflectance spectrum from the wing sample of the *Greta oto* butterfly at 45° and Bragg curves for m = 1 (black line), m = 2 (red line) and m = 3 (green line). (**e**) Photonic dispersion relation for the 2D model; the inset shows the periodic array observed in the electron microscopic image. (**f**) PDoS for the TE mode for the PBG shown in (**e**). (**g**) Photonic band structure for the theoretical model of the *G. oto* butterfly wing with the chitin-air arrangement. (**h**) PDoS for the TE mode for the PBG shown in (**g**) with cone and cylinder arrays. (**i**) Cone and cylinder array spatial variation of the dielectric function of the simulated system for the top point of view.

Using the electron microscopic image and FT results, the structures observed in the two samples are modeled to evaluate the photonic dispersion relation. The structures in the wings of the *M. cypris* butterfly were modeled similar to 1D photonic crystals composed of a multilayer of alternating chitin and air. A schematic representation of the spatial variation in the dielectric function can be observed in the inset of Fig. 10(c), where blue (white) zones represent layers of chitin (air). Chitin is the predominant compound in butterfly wings^12^. The lattice parameter is ~0.63 μm, while the chitin layer thickness d_chitin_ = 0.51 μm and the air section thickness d_air_ = 0.12 μm (Fig. 3(d), 7(b)). Photonic band gaps (PBGs) are associated with light reflections because an important key is to compare the reflectance spectrum and the calculated photonic band structure (Fig. 10).

Using the SEM image of the *G. oto* sample, the spacing distribution between the structural elements present was assembled according to the measurements made. The distribution of inter-structural features between the reliefs is shown in Fig. 8. This distribution has a maximum centered at 223 nm and a half-maximum width of 70 nm. The fact that the distribution of distances is much narrower than the average distance between the reliefs shows that a large degree of periodicity is involved. It could be said that the ratio between the variance in the distribution and the most expected (maximum) value may reveal a certain degree of periodicity. A unit value of this ratio represents full randomness, while a zero value represents a perfect periodic structure.

For the present case, we have a factor of approximately 0.3, which is closer to a periodic behavior than to a random behavior. In addition to the site-to-site analysis, the size distribution of the present structural aspects is also important from the light scattering point of view. The analysis of the sizes and their distribution are shown in Fig. 9. Here, we also note the size distribution, with the most expected value being 133 nm and a half-height distribution width on the order of 40 nm. Once more, from a structural size point of view, the periodicity factor is approximately 0.3, again corroborating that the sturdiness is more periodic than random.

All these facts, however, allow us to conclude that the analysis indicates that the considered structure is more periodic than random, ensuring the applicability of the band analysis techniques for these cases.

The results of a similar study for the wings of the Greta oto butterfly can be seen in Fig. 4(a,b), corresponding to the same resolutions as in the previous case. In Fig. 4(a), no macroscopic structures are observed that suggest a periodic pattern, except for the presence of filament-like scales or hairs. By increasing the resolution, i.e., Fig. 4(b), it is possible to distinguish an aggregate of points, or even triangular shapes, that suggests an organization in 2 dimensions. Even in some areas, it is possible to identify a hexagonal pattern with an average diameter of 130 nm and a network parameter on the order of 400 nm.

For the structures observed in the wings of the Greta oto butterfly, the first idealized model is used, a hexagonal network formed by a distribution of air cylinders immersed in a chitin matrix. Despite being a toy model regarding experimental observation, it sheds light on the photonic behavior of the structures observed.

The reflectance spectrum from the wing sample of the *M. cypris* butterfly at 30° and the Bragg curves are shown in Fig. 10(a) and are compared with the 1D photonic dispersion relation (Fig. 10(b)). The photonic band structure in the present model is well known and is characterized by degenerated transverse electric (TE) and magnetic (TM) modes. There is a band gap between 6.43 and 6.8 × 10^14^ Hz, which matches very well with the experimental blue peak, the main color that is observed directly in the wings. This feature is confirmed by the photonic density of states for the TE mode for the PBG obtained, as shown in Fig. 10(c). Despite the inherent disorder present, as shown in Fig. 3(d), the photonic band structure was evaluated by assuming an ideal photonic crystal.

In a similar way, structures found in the sample of the *G. oto* butterfly wing were modeled as 2D photonic crystals (Fig. 10(e,g)). In particular, we first modeled a chitin matrix with a hexagonal distribution of identical circular air holes (Fig. 10(f,i)). The electron microscopic image in Fig. 7(a) shows an average diameter d = (0.24 ± 0.03) μm for the holes and a lattice parameter a = (0.43 ± 0.03) μm for the hexagonal lattice. The reflectance spectrum at an angle of 45° from Fig. 2(b) and the Bragg curves and photonic density of states for the TE mode are compared with the photonic dispersion relation in Fig. 10(d–f). In this case, the PBG presents TM and TE modes that have not degenerated, and the dispersion relations exhibit different slopes for different bands and directions of the wave vector k. Additionally, frequency gaps are not found in the visible region of the spectrum (Fig. 10(e–h)).

Although iridescence has been related to periodic structures^27^, it is possible to show that this phenomenon is more complex. The light–matter interaction can produce a variety of optical phenomena, such as diffuse reflection, specular reflection, diffraction or even Bragg interference when matter is organized into periodic arrangements. When the wavelength of light is the same order of magnitude as the lattice parameter, Bragg reflection can be possible^28^. In fact, Bragg’s law makes possible many devices routinely used in tests in medicine, physics, and biology, among other areas. An important characteristic of Bragg’s law, mλ = 2a sinθ, is that it does not depend on the system dimensions^29^. In this equation, *m* is a positive integer, *λ* is the wavelength of the incidental light, *a* is the lattice parameter and *θ* is the angle of incidence of light.

Bragg’s equation was employed to obtain the frequency behavior as a function of the angle for both the 1D model and 2D model. For *M. cypris*, it is shown in Fig. 10(a), joined with the frequency curves for *m* = 1 (black line), *m* = 2 (red line) and 3 (green line); there is a reflectance curve for θ = 30° (*M. cypris*). Figure 10(a) reveals that by Bragg’s law, the blue color could be observed only at θ = 20° at the first order (*m* = 1) and around θ = 45° at the second order (*m* = 2). The reflectance spectra in Fig. 2(a) show that the color blue can be seen at other angles.

Similarly, Fig. 10(d) shows the results for the *G. oto* model, where the green line presents the reflectance spectra at 45° (from Fig. 2(b)), and it has peaks at 465 nm, 540 nm, 590 nm and 620 nm. The Bragg curves show that every color occurs at only one specific angle and, for *m* = 1, between 20° and 70°. For other values of *m*, there are no observable colors. Again, the experimental results disagree with the theoretical predictions. From previous discussions, Bragg’s reflection is apparently not enough to explain the phenomenon of iridescence in the two present cases.

In conclusion, our results suggest a strong correlation among the periodic nanostructures found on the surface of the wings and the iridescence effect exhibited for *M. cypris* and *G. oto* Colombian butterflies. In particular, the results of the structural-based color in the *M. cypris* butterfly confirm the recent observation of Kinoshita, S., & Yoshioka, S^2^. These nanostructures are 1D or 2D photonic crystal-like structures, and they can inspire the design of novel photonic devices, even the manufacturing of makeup and cosmetic or industrial paints.

## Methods

### Experimental

**Figure 11:**
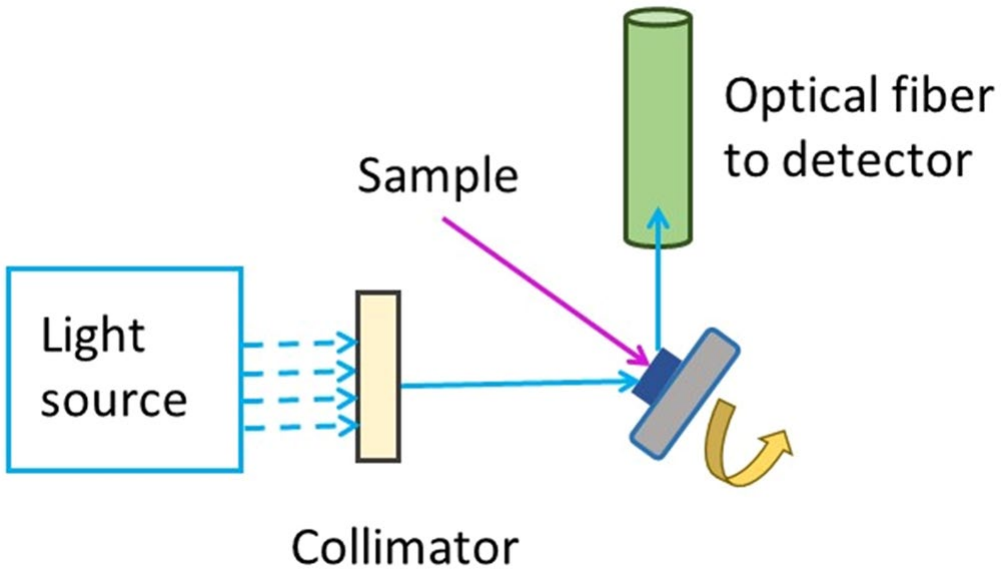
Schematic of the experimental setup implemented to develop the reflectance measurements. The angle was varied at an interval of five grades from 0° to 90° for both butterfly samples.

The *M. cypris* and *G. oto* structures were explored via scanning electron microscopy (SEM) measurements, which were performed by using a microscope (VEGA3 SB) with a tungsten filament, an accelerating voltage of 4.89 kV in vacuum conditions (10^−6^ mbar), and an XFlash Detector 410 M. The absorption and transmittance spectra were obtained using a Cary 50 Bio UV-Vis Spectrophotometer; the baseline was taken with a quartz cuvette. The fluorescence measurement of the samples was acquired in the range of 350 nm to 1000 nm for an integration time of 200 ms with a portable USB-2000 spectrometer (Ocean-Optics USA) with a computer and two 600 μm optical fibers, one of which acts as the source, while the other one, as a collector. A nonfluorescent base and two lasers of wavelength 408 nm and 532 nm with a longpass filter at 550 *nm* (OGG550-Schott-USA) were used. The data were collected with the help of the OBase32.exe software (Ocean Optics). The reflectance measurements were performed by varying the angle at intervals of five grades, from 0° to 90°, with an optical fiber and an LED source of white light. The data were acquired with an optical fiber and the portable USB. The schema of the experimental setup is shown in Fig. 11. The Zeiss LSM 780 inverted fluorescent confocal microscope was used to obtain the optical details of the wing structure thanks to a magnification of 200, with the channels of the wavelength ranging from 415–599 nm and from 599–758 nm. The autofluorescence measurement was obtained via a laser with a wavelength of 405 nm, and light transmission was evidenced.

Figure 5 shows the experimental optical microscope setup used to take pictures of both butterflies without filters. Figure 6 shows the photographs obtained with this setup for different azimuth angles relative to the *M. cypris* butterfly wings. These photographs were taken with a commercial cyber-shot SONY camera of 14.1 megapixels.

### Theory

The principal characteristic of photonic crystals is the spatial periodic variation in the dielectric function^30–32^. This behavior originates from photonic band structures after light interacts with them. A partial or total gap^30^ is presented to the transverse electric (TE) and transverse magnetic (TM) components of the field. Using Block theorem^33^, the introduction of a harmonic description of the electric, $\overrightarrow{E}(\overrightarrow{r},t)$, and magnetic, $\overrightarrow{H}(\overrightarrow{r},t)$, fields in Maxwell’s equations is performed to describe the propagation of light in nature, and the mathematical development of the master equation yields^30–32^:

1 $$\overrightarrow{{\nabla }}\times \left[\frac{1}{{\epsilon }(\overrightarrow{r})}\overrightarrow{{\nabla }}\times \overrightarrow{H}(\overrightarrow{r})\right]={\left(\frac{\omega }{c}\right)}^{2}\overrightarrow{H}(\overrightarrow{r})$$

with $\omega =\omega (\overrightarrow{k})$ being the wave vector $\overrightarrow{k}$. The eigenvalues, $\omega (\overrightarrow{k})$, are the permitted frequencies of the system, i.e., the modes.

From the electron microscopic image in Fig. 7 combined with the identification of the ordered structure from image analysis, the computational modeling was constructed. Taking into account that the butterfly wings have a dielectric array and are spatially varying, we modeled the systems similar to a 1D or 2D photonic crystal. The photonics band calculatio™ns were obtained with the free MIT photonics bands (MPBs)^34^.

## Supplementary information

Supplementary information 2.

Supplementary information 3.

Supplementary information 4.

Supplementary information 5.

Supplementary information 6.
