# Supplementary figures and images for: Photonic effects in natural nanostructures on Morpho cypris and Greta oto butterfly wings
Source: Sci Rep. 2020 Apr 1;10:5786. doi: 10.1038/s41598-020-62770-w (PMC7113256; doi:10.1038/s41598-020-62770-w)

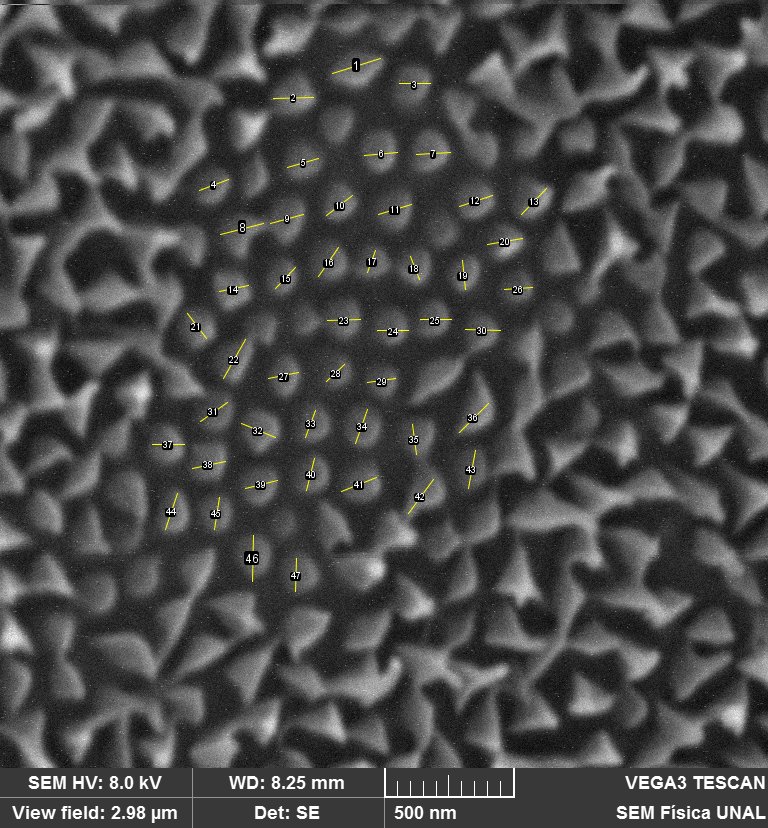

Supplement: Supplementary file 2 — Supplementary information 3. [file 41598_2020_62770_MOESM2_ESM.jpg]

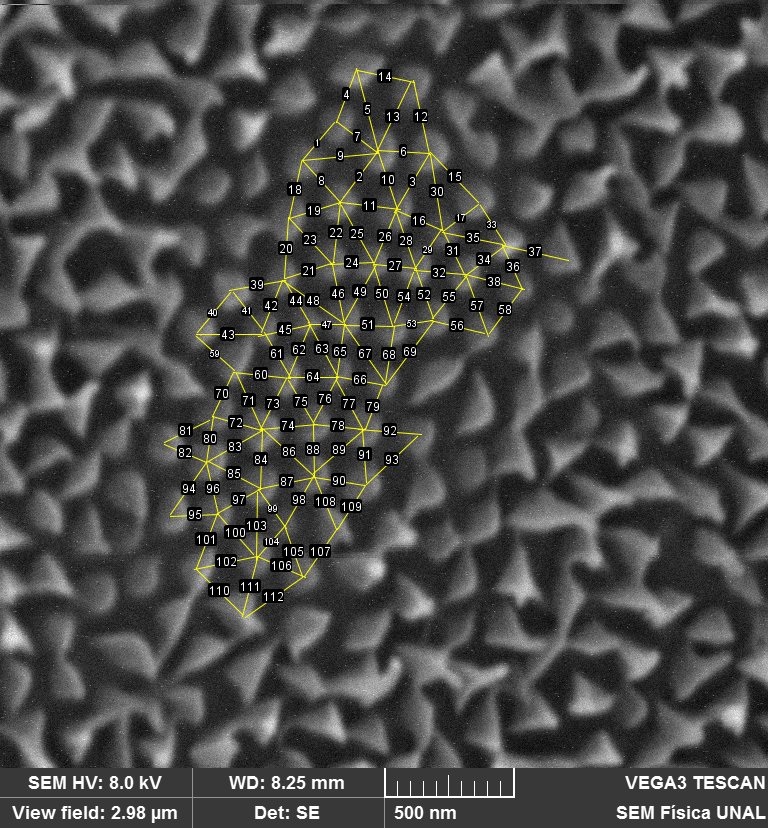

Supplement: Supplementary file 4 — Supplementary information 5. [file 41598_2020_62770_MOESM4_ESM.jpg]
